# The impact of mass drug administration and long-lasting insecticidal net distribution on *Wuchereria bancrofti *infection in humans and mosquitoes: an observational study in northern Uganda

**DOI:** 10.1186/1756-3305-4-134

**Published:** 2011-07-15

**Authors:** Ruth A Ashton, Daniel J Kyabayinze, Tom Opio, Anna Auma, Tansy Edwards, Gabriel Matwale, Ambrose Onapa, Simon Brooker, Jan H Kolaczinski

**Affiliations:** 1 Malaria Consortium Africa, Kampala, Uganda; 2 Department of Disease Control, London School of Hygiene and Tropical Medicine, London, UK; 3 Malaria Consortium Uganda, Kampala, Uganda; 4 District Health Office, Dokolo, Uganda; 5 Vector Control Division, Ministry of Health, Kampala, Uganda; 6 MRC Tropical Epidemiology Group, London School of Hygiene and Tropical Medicine, London, UK; 7 RTI International, Kampala, Uganda; 8 Malaria Public Health & Epidemiology Group, Kenya Medical Research Institute-Wellcome Trust Research Programme, Nairobi, Kenya

## Abstract

**Background:**

Lymphatic filariasis (LF) in Uganda is caused by *Wuchereria bancrofti *and transmitted by anopheline mosquitoes. The mainstay of elimination has been annual mass drug administration (MDA) with ivermectin and albendazole, targeted to endemic districts, but has been sporadic and incomplete in coverage. Vector control could potentially contribute to reducing *W. bancrofti *transmission, speeding up progress towards elimination. To establish whether the use of long-lasting insecticidal nets (LLINs) can contribute towards reducing transmission of *W. bancrofti *in a setting with ongoing MDA, a study was conducted in an area of Uganda highly endemic for both LF and malaria. Baseline parasitological and entomological assessments were conducted in 2007, followed by high-coverage LLIN distribution. Net use and entomological surveys were carried out after one year, and final parasitological and entomological evaluations were conducted in 2010. Three rounds of MDA had taken place before the study commenced, with a further three rounds completed during the course of the study.

**Results:**

In 2007, rapid mapping indicated 22.3% of schoolchildren were *W. bancrofti *antigen positive, and a baseline survey during the same year found age-adjusted microfilaraemia prevalence was 3.7% (95% confidence interval (CI): 2.6-5.3%). In 2010, age-adjusted microfilaraemia prevalence had fallen to 0.4%, while antigenaemia rates were 0.2% in children < 5 years and 6.0% in ≥ 5 years. In 2010, universal coverage of mosquito nets in a household was found to be protective against *W. bancrofti *antigen (odds ratio = 0.44, 95% CI: 0.22-0.89). Prevalence of *W. bancrofti *larvae in anopheline mosquitoes had decreased significantly between the 2007 and 2010 surveys, but there was an apparent increase in vector densities.

**Conclusion:**

A marked reduction in *W. bancrofti *infection and infectivity in humans was observed in the study area, where both MDA and LLINs were used to reduce transmission. The extent to which LLINs contributed to this decline is equivocal, however. Further work investigating the impact of vector control on anopheline-transmitted LF in an endemic area not benefitting from MDA would be valuable to determine the effect of such interventions on their own.

## Background

Lymphatic filariasis (LF) is a major cause of acute and chronic morbidity of humans living in the tropics. The disease is caused by infection with the parasitic worm *Wuchereria bancrofti *in Africa (*Brugia malayi *and *B. timori *in Asia-Pacific) and is transmitted by *Anopheles, Culex, Aedes *and *Mansoni *mosquitoes [1,2]. Infection with the parasite leads to disabling lymphoedema, hydrocoele and acute adenolymphangitis (ADL) [3,4], as well as less-evident morbidity such as adenopathy, lymphangitis, haematuria and proteinuria [3,5,6]. LF is currently endemic in 81 countries and, while it has been largely controlled in much of the Pacific and China, remains an important health problem in India and sub-Saharan Africa [7].

LF is targeted for elimination by the year 2020 [8], with the primary tool to achieve this ambitious goal being mass drug administration (MDA) of albendazole plus either ivermectin or diethylcarbamazine citrate (DEC) to the at-risk population [9]. Regular MDA reduces microfilaria (mf) loads and hence LF transmission, but has little macrofilaricidal effect [10]. MDA therefore needs to continue until adult worms lodged in human hosts die naturally or cease to be fecund (mf producing), which has been estimated to take about five years [11]. Maintaining high coverage of MDA over several years is challenging, particularly in resource constraint settings [12], and has to proceed with caution or not at all in areas where the parasitic worm *Loa loa *is prevalent, due to the increased risk of severe adverse events [9,13]. Due to these limitations of MDA, vector control is increasingly being considered as a complementary tool for LF elimination [14-16], particularly in locations where *Anopheles *mosquitoes are the vector and malaria is co-endemic.

The naturally inefficient transmission of *W. bancrofti*, as well as the process of facilitation, whereby development of infective L3 larvae becomes more efficient with increased numbers of *W. bancrofti *ingested by the mosquito, but consequently reduces mosquito survival [17,18], make vector control particularly attractive for LF control. Perhaps the key factor supporting use of vector control against LF is the ongoing scale-up in long-lasting insecticidal net (LLIN) coverage against malaria in sub-Saharan Africa [19]. *W. bancrofti *is transmitted by *Anopheles *across west Africa and in some rural areas of east Africa. In these areas co-endemic for malaria and *Anopheles-*transmitted *W. bancrofti*, use of LLINs has the potential to successfully reduce transmission of both parasites, but despite promising results from pilot studies indicating potential benefits of LLINs against LF (Table 1) [20-23], longitudinal data to this effect are few [24].

**Table 1:** Summary of current evidence of describing impact mosquito nets on *W. bancrofti *transmission by Anopheles

Key findings	Vector	Location	Reference
LLIN distribution targeted to pregnant women and < 5s resulted in reduction of 60% in vector population Universal LLIN coverage resulted in 90% decline in vector population Both LLIN strategies resulted in significant reduction in proportion of mosquitoes with any *W. bancrofti *larval stage	*Anopheles *spp.	Nigeria	[24]

Use of insecticide-treated net (ITN) reduced density of indoor resting *An. gambiae s.l*. and *An. funestus* *An. funestus *and *An. gambiae *showed slight switch to animal feeding after ITN adoption, *Cx. quinquefasciatus *switch to majority animal feeding Vector potential reduced by ≥ 97% for *Cx. quinquefasciatus, An. gambiae *and *An. funestus*	*An. gambiae*, *An. funestus*, *Cx. quinquefasciatus*	Kenya	[20]

Introduction of ITNs resulted in reduction of overall mosquito density by 22.6% Annual transmission potential was reduced by 92% Annual infective biting rate was reduced by 95%	*An. funestus*, *An. gambiae*, *Cx. quinquefasciatus*	Kenya	[21]

In case-control study, individuals using untreated mosquito net had higher odds of LF than those using ITNs	Not specified	Cambodia	[22]

Use of untreated mosquito nets was associated with reduced odds of *W. bancrofti *antigenaemia and microfilaraemia Individuals using untreated mosquito nets also had lower odds of hydrocoele than non-users	*An. farauti*, *An. punctulatus*, *Cx. annulirostris*, *Cx. quinquefasciatus*	Papua New Guinea	[23]

LF in Uganda is caused by *W. bancrofti *and transmitted by *Anopheles gambiae s.l*. and *An. funestus *mosquitoes [25]; the disease is largely concentrated in the north of the country [26]. MDA with albendazole and ivermectin commenced in 2002 [12], but has been sporadic and incomplete [27]. Since 2007, concerted efforts have been undertaken to expand MDA coverage to target 7.2 million people [28]. Coverage of malaria-endemic areas with LLINs has been steadily increasing [29] and the goal of the national policy has recently been amended to focus on universal coverage (defined as one net for every two people) [30].

The aim of the present work was to investigate whether large-scale distribution of LLINs could contribute towards the MDA-based transmission control of LF. Specifically, this included estimating prevalence of *W. bancrofti *infection pre- and post-intervention with LLIN distribution, and investigating associations between *W. bancrofti *infection and various indicators of ownership and use of mosquito nets.

## Methods

### Site selection

**Figure 1:**
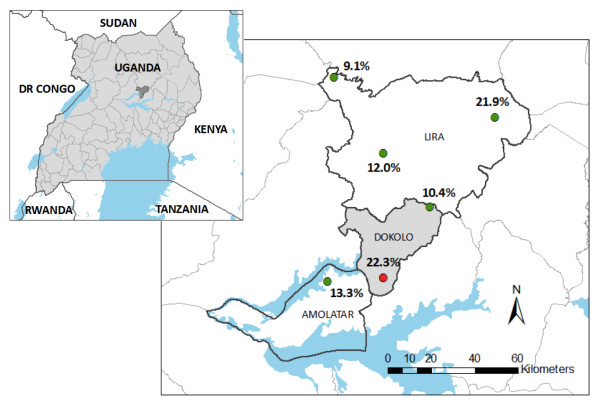
**Results of rapid mapping at six primary schools in Northern Uganda in May 2007: prevalence of *W. bancrofti *antigen in children aged 6-16, determined by immunochromatographic test (Binax Now filariasis, Portland, ME)**.

In May 2007, rapid LF mapping was conducted using immunochromotographic tests (ICT; BinaxNOW Filariasis, Binax Inc., Scarborough, ME) to detect *W. bancrofti *antigen in school children, according to World Health Organization (WHO) recommendations [9]. Children from six primary schools in Lira, Amolatar and Dokolo districts, north of lake Kyoga, were tested (Figure 1). These districts had previously been identified as highly endemic for *W. bancrofti *[26]. Selection of schools for rapid mapping was based on a sampling grid with 50 km between each school [9,31], as well as anecdotal reports of high infection levels provided by the District Health Offices. At each school, 175 children aged between six and 16 years were randomly selected to provide a finger prick blood sample for analysis by ICT.

The school in Adeknino parish, Dokolo district, had the highest prevalence of *W. bancrofti *antigen (22.3%, 95% confidence interval (CI) 16.4-29.2%). Seven adjacent villages in Adeknino parish were therefore selected as the study area for detailed parasitological surveys, which was deemed sufficient to reach the calculated sample size.

### Study design

**Figure 2:**
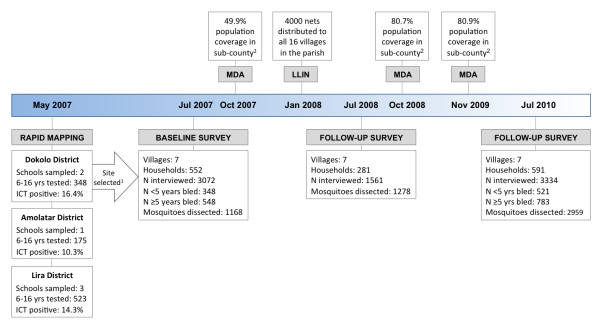
**Flowchart describing study activities 2007-2010 and interventions conducted at the study site including mass drug administration with ivermectin and albendazole (MDA) against *W. bancrofti*, and distribution of long-lasting insecticide-treated mosquito nets (LLINs)**. ^1 ^The school with the highest *W. bancrofti *prevalence as determined by ICT, was in Adeknino Parish in Dokolo. A group of seven adjacent villages in this parish were selected as the study site. ^2 ^MDA population coverage calculated as number of individuals receiving albendazole and ivermectin, divided by the projected total population of the sub-county which includes Adeknino Parish.

A pre- and post-intervention observational study was conducted to examine the impact of MDA and mass distribution of LLINs. A timeline of study activities, including delivery of interventions, is shown in Figure 2. Parasitological surveys to determine *W. bancrofti *microfilariaemia were conducted in July 2007 and July 2010. Rates of *W. bancrofti *antigenaemia were also evaluated in 2010 using ICTs. The survey in July 2008 did not include collection of blood samples. During all three surveys, three key mosquito net variables were evaluated: i) individual use of a net on the previous night, ii) number of nets in the household, and iii) universal coverage with nets in the household. In addition, *Anopheles *mosquitoes were caught and dissected during each survey, to estimate the prevalence of *W. bancrofti *infection and infectivity in the vector population. MDA with albendazole and ivermectin were delivered in October 2007, October 2008 and November 2009. Four thousand LLINs were distributed to all 16 villages in Adeknino Parish in January 2008.

### Baseline survey

A population census in the study area was carried out in July 2007 using a standardised questionnaire. All households in the seven villages were visited; verbal consent for inclusion was obtained after explaining the purpose of the interview. Basic demographic details of household members and key socioeconomic characteristics were recorded, as well as information on mosquito net ownership and use. Mosquito net ownership and condition was verified by visual inspection. In order to capture the protection conferred by LLINs in different age groups, two individuals, one below and one above five years of age (with no upper age limit), were randomly selected from each household by roll of a die, and requested to provide a night-time blood sample for detection of *W. bancrofti *microfilaria.

Upon arrival at a central location between 9 pm and 1 am, participants were informed of procedures and requested to provide verbal consent. Participants were examined by a medical doctor for signs of elephantiasis and hydrocoele according to standard WHO definitions [32]; the same doctor performed all clinical examinations. A single finger-prick blood sample was used to prepare a thick blood smear, and to collect 100 μl blood using a heparin-coated capillary tube that was added to 900 μl 3% acetic acid for microfilaria detection by counting chamber technique.

### LLIN distribution follow-up survey

A follow-up survey was conducted in July 2008, six months after distribution of 4,000 LLINs to the 16 villages in the study parish. Forty households from each of the seven study villages were selected by random walk, beginning from the centre of the village where a pen was spun to select the direction. The head of household was interviewed using a standardised questionnaire to determine basic household characteristics, socio-economic indicators and use of mosquito nets by household members. Condition and location of all mosquito nets in the household were verified by visual inspection.

### Final follow-up survey

Three years post-baseline (July 2010), a final population census and parasitological survey was conducted. Since the prevalence of *W. bancrofti *mf found at baseline was lower than assumed in the sample size calculation, the sampling strategy was adjusted to maximise power to determine a change in mf prevalence. It was decided to over-sample children under five years during the parasitological survey to generate sufficient data to allow estimation of recent transmission. Therefore, all children under five years of age in the study villages were included in the survey. Furthermore, a stepwise diagnostic approach was taken, with ICTs being used as the first-line tool, followed by night bleeds only for ICT-positive individuals. This approach took advantage of the high sensitivity of ICTs for detection of *W. bancrofti *infection, hence minimizing the logistical difficulties involved in conducting large numbers of night bleeds.

All households in study villages were visited and a standardised questionnaire carried out, similar to the questionnaire used at baseline. At all households, a finger-prick blood sample was collected from any child below five years of age for preparation of ICT test. At every third household visited, household members of all ages were invited to be included in blood sampling. Any individual with a positive ICT result was requested to attend night-time blood sampling between 9 pm and 1 am, where a thick blood film was prepared and 100 μl blood stored in 900 μl 3% acetic acid.

### Entomological surveillance

Entomological surveillance was conducted at the same time as household surveys in 2007, 2008 and 2010. A CDC light trap was set next to a sleeping place in ten randomly selected consenting households at dusk each day. Untreated mosquito nets were provided for all sleeping places in buildings where a light trap was set, and any hanging insecticide-treated nets removed while the traps were in use. Traps were collected at dawn the following day. Mosquitoes were anaesthetised, sorted by sex and species, and female *Anopheles *were dissected and mounted on slides. Light traps were set in ten new households every night until the target sample size of *Anopheles *was reached: 1,000 in 2007 and 2008, and 3,000 in 2010.

### Microscopy

Thick blood films were air-dried and stained with Giemsa, then examined for presence of *W. bancrofti *microfilaria. Acetic acid blood samples were examined for *W. bancrofti *by counting chamber method, and the number of microfilaria recorded and converted to microfilaria per ml of peripheral blood (mf/ml) [33]. Dissected mosquitoes mounted on slides were fixed with methanol, then stained using haematoxylin stain and examined for filarial species. The species and number of larvae, developmental stage and location were recorded.

### Sample size

Sample size calculations were conducted in Stata 9.2 (Stata Corporation, College Station, TX) using the 'sampsi' command, aiming at 80% power and a significance level of 5%, exploring a variety of scenarios with estimated *W. bancrofti *prevalence ranging from 10-18% and protective efficacy of LLINs from 27-55%. These calculations indicated that 1,000 individuals would need to be sampled at each time-point to determine whether there was a statistical difference in prevalence of *W. bancrofti *infection between individuals protected by a LLIN and those individuals unprotected. No allowances for inter-village variation were made, since it was assumed that the population and infection risk in the selected villages would be similar. It was not possible to conduct the study in a single village of Adeknino parish due to small village populations; therefore seven adjacent villages were included.

Sample size for entomological surveys was calculated assuming 6% of dissected mosquitoes would be infected [25], and a reduction of 50% in proportion infected would be seen, with 80% power and significance level of 5%. However, since prevalence of infection was found to be lower than expected at baseline, the sample size was increased in the final follow-up survey to the maximum number logistically feasible to collect and dissect.

### Analysis

Data were entered into a Microsoft Access database (Microsoft corporation, Seattle, WA) subject to validation checks. Statistical analysis was carried out in Stata 9.2. Principal component analysis was used to create a wealth index for each household that adequately summarised socio-economic factors; household were then divided into quintiles according to this index [34].

Mosquito net use and ownership were measured and analysed in the following ways: i) sleeping under a net on the evening prior to the survey (categorical: no net used, slept under untreated net, slept under LLIN); ii) universal net coverage in household, a binary variable measured as one or more nets for every two individuals, iii) number of nets per household (discrete). To quantitatively assess the condition of existing nets, a proportional hole index was calculated, where number and size of holes in each net are summarised. This is calculated as the number of finger-sized holes, plus number of fist-sized holes multiplied by nine, plus number of head-sized holes multiplied by 56. A proportional hole index of < 25 was categorised as good, 25-299 as moderate, and ≥ 300 as poor condition (A. Kilian, personal communication).

Coverage data for each round of MDA were provided by central records of the national LF control programme (2002-2006 data) and Dokolo district health office (data for 2007 onwards). Coverage was defined as number of individuals receiving ivermectin and albendazole, divided by the projected total population as calculated by the Uganda Bureau of Statistics. Coverage data for 2007-2009 are presented at the sub-county level, while data for 2002, 2003 and 2005 are presented as total for the former Lira district, which includes the current Lira, Dokolo and Amolatar districts.

Unadjusted prevalence of *W. bancrofti *mf is presented for children under five years and all those aged five and older from 2007 and 2010 surveys, as well as *W. bancrofti *antigen prevalence from 2010. Exploratory analysis using generalised linear and latent mixed models (GLLAMM) found little evidence for any effect of clustering of *W. bancrofti *mf or antigen by village. Simple generalised linear models (GLM) were therefore used to produce age-adjusted prevalence estimates and corresponding 95% CI for *W. bancrofti *mf in 2007 and 2010, as well as *W. bancrofti *antigen in 2010. Each GLM was tested with both linear and categorical (ten-year bins, then all ≥ 50) age variables to determine any departure from linear trend. All the GLM included sex *a priori*, and were calculated using robust standard error estimates. Chi squared test was used to compare crude and adjusted prevalence of infection between baseline and follow-up.

To investigate associations between each infection outcome (*W. bancrofti *antigen and *W. bancrofti *mf) and net use and ownership measures at three years post-baseline across all ages, logistic regression was first used to test each net ownership and use measure and socio-demographic characteristics in univariate analyses. The net variables considered key primary exposures for separate multivariate models were a categorical variable of sleeping under a net on the previous night (did not sleep under a net, slept under untreated net, slept under LLIN) and universal coverage with any net type in the household. In multivariable modelling, age category, sex and village-level baseline *W. bancrofti *mf prevalence among those aged five and older were included in all models *a priori*. A final model was obtained using a forward stepwise approach with inclusion criteria of p < 0.1, based on results of likelihood ratio tests (LRT), testing covariates with strongest univariate association with outcome first. Robust standard errors were applied to final multivariate models, to adjust for potential underlying heterogeneities in the data and therefore provide a conservative estimate of association between net indicators and outcome.

Density of *Anopheles *mosquitoes was described using the monthly biting rate (MBR) for July, calculated as: (total mosquitoes caught*31)/(number of traps*number of nights traps set).

The MBR assumes that the number of mosquitoes caught in a light trap is equivalent to that which would be collected by human landing catches. Monthly transmission potential (MTP) describes the mean number of *W. bancrofti *infectious bites received during the month, calculated as: (MBR*number mosquitoes with L3 infection)/total mosquitoes caught. The proportion of all mosquitoes carrying any *W. bancrofti *larval stage is also presented.

### Ethical considerations

The Uganda National Council for Science and Technology approved the study protocol (HS 271). Clearance to conduct the surveys was obtained from the local authorities. Verbal informed consent to conduct interviews was received from head of household and recorded on questionnaires. Verbal consent was requested from all adults and parents or guardians of minors for collection of blood samples, with approval given/refused recorded on consent forms. Individuals that declined to participate were excluded from the study. Verbal informed consent was gained from head of household before setting light traps, and untreated mosquito nets were provided for all sleeping places in each structure where traps were set.

## Results

### Study population

The number of households and individuals interviewed and providing blood samples in the 2007, 2008 and 2010 surveys are detailed in Figure 2. In 2010, 43/48 (90%) of individuals with a positive ICT result provided a night bleed sample. In the 2007 and 2010 surveys, individuals who had been resident in the sub-county for less than two years were excluded (n = 144 in 2007, n = 169 in 2010), while families that had been resident in the village for fewer than seven months, the time since the LLIN distribution, were excluded from the 2008 survey (n = 6).

### Mosquito net ownership and use

In 2007, households had a mean number of 1.0 nets, with only 14.5% of households found to have sufficient nets to allocate one to every two people (defined as universal coverage). Of all nets examined in 2007, 50.5% were LLINs and 57.9% were in good condition. Six months after the LLIN distribution in 2008, households had an average of 2.8 nets, and 55.5% had achieved universal coverage. Unsurprisingly, 94.7% of nets were in good condition and 91.0% of all nets were in use (hanging up over sleeping places) shortly after the distribution. By 2010, net coverage and condition had deteriorated to a mean of 2.0 nets per household, with only 38.1% households being in the universal coverage category. Only 36.8% of nets remained in good condition. The main reason given by individuals for discarding their nets was that they had developed holes.

At baseline in 2007, 43.3% of all individuals reported sleeping under a mosquito net on the previous night. After the LLIN distribution, this rose to 81.9% of people sleeping under a mosquito net, but in 2010 only 60.2% of those surveyed reported using a net during the previous night. Summary net use stratified by age and net type are presented in Table 2.

**Table 2:** Key indicators of mosquito net and LLIN ownership and use over the study period

	2007	2008	2010
Number of households assessed	552	281	591
Number of individuals assessed	3072	1569	3334
Mean nets per household	1.0 (0.9-1.1)	2.8 (2.7-2.9)	2.0 (1.9-2.1)
Households with universal net coverage^1^	14.5 (11.7-17.7)	55.5 (49.5-61.4)	38.1 (34.1-42.1)
Percent of nets found hanging or tied over sleeping place	93.1 (90.6-95.1)	90.6 (88.2-92.6)	83.5 (81.3-85.6)
Percent of nets being LLINs	50.5 (45.6-55.3)	95.3 (93.6-96.7)	86.7 (84.7-88.6)
Mean LLINs per household	0.5 (0.4-0.5)	2.5 (2.4-2.7)	1.7 (1.6-1.8)
Net use on previous night, among < 5 years:			
Did not sleep under a net	64.6 (60.8-68.4)	11.4 (8.2-15.4)	27.1 (23.7-30.8)
Slept under untreated net	14.6 (12.0-17.7)	8.3 (5.6-11.9)	17.9 (15.0-21.2)
Slept under LLIN	20.7 (17.6-24.1)	80.2 (75.5-84.4)	54.9 (50.9-58.9)
Net use on previous night, among ≥ 5 years:			
Did not sleep under a net	72.5 (70.7-74.3)	19.6 (17.4-21.9)	42.8 (40.9-44.7)
Slept under untreated net	14.8 (13.4-16.3)	8.6 (7.1-10.3)	9.0 (7.9-10.1)
Slept under LLIN	12.7 (11.4-14.1)	71.8 (69.2-74.3)	48.2 (46.3-50.1)

Data are percent (exact binomial 95% confidence intervals) unless otherwise stated.

^1^One mosquito net for every two people in the household

### Mass drug administration

MDA with ivermectin and albendazole began in Uganda 2002, targeting two districts (including the study site) out of the total 22 LF endemic districts. MDA was scaled up gradually in the following years, but no MDA took place during several years due to lack of operational funds [27]. Annual MDA has routinely taken place since 2007 (Figure 2), usually in October each year during Child Health Days Plus, but occasionally delayed due to financial or logistical constraints. The villages in the study had received six rounds of MDA by the end of the study; in 2002, 2004-5 and 2007-9, with reported population coverage between 49.9% and 80.9%.

### *W. bancrofti *infection in humans

In the baseline survey conducted in all seven study villages, the sex- and age-adjusted (referred to as age-adjusted from this point) *W. bancrofti *mf prevalence was 3.7%. Crude mf prevalence in children under five years was 0.9%, and 6.8% in those aged five years and above. The geometric mean mf intensity was 165 mf/ml (95% CI 98-277 mf/ml, Table 3).

**Table 3:** Key indicators of *W. bancrofti *in human population over the study period.

	2007	2010
Number of individuals providing blood samples	896	1304
Number of blood samples from < 5 yrs (%)	348 (38.9)	521 (40.0)
Crude % prevalence estimates^1^		
*W. bancrofti *antigen in < 5 years	-	0.2 (0.01-1.1)
*W. bancrofti *antigen in ≥ 5 years	-	6.0 (4.4-7.9)
*W. bancrofti *mf in < 5 years^2^	0.9 (0.2-2.5)	0.0 (0.0-0.7)^3^
*W. bancrofti *mf in ≥ 5 years^2^	6.8 (4.8-9.3)	0.8 (0.3-1.7)
*W. bancrofti *geometric mean (range)	165 (10-3,790)	80 (10-260)
Lymphoedema/elephantiasis	1.2 (0.6-2.2)	-
Hydrocoele, amongst males	1.5 (0.6-3.3)	-
Acute adenolymphangitis	1.2 (0.6-2.2)	-
Adjusted % prevalence estimates^4^		
*W. bancrofti *antigen	-	2.1 (1.2-3.7)
*W. bancrofti *mf	3.7 (2.6-5.3)	0.4 (0.2-1.0)

^1 ^With exact binomial 95% confidence interval

^2 ^All ICT negative samples assumed to be mf negative

^3 ^One-sided test, 97.5 confidence interval

^4 ^Estimated using generalised linear models, with robust 95% confidence intervals. All models are also adjusted for sex. Age was included as a linear variable in *W. bancrofti *mf 2007 and 2010 models. Age was included as a categorical variable with 10 year bins and grouping all ≥ 50 years in *W. bancrofti *antigen 2010 model.

Acute adenolymphangitis (ADL) was diagnosed in 1.2% individuals of all ages examined at baseline. A further 1.2% had some form of elephantiasis: the leg was most commonly affected, but single cases in the arm, breast and scrotum were identified. Six cases of hydrocoele were diagnosed (1.5% males of all ages), with four having previously received corrective surgery.

The age-adjusted *W. bancrofti *antigen prevalence in 2010 was 2.1% (Table 3). The unadjusted prevalence of *W. bancrofti *antigen in children under five years was 0.2%, and 6.0% among those aged five and older (Table 3). The youngest ICT positive individual in 2010 was four years old. Assuming that the ICT provides a sensitive marker of infection, the age-adjusted mf prevalence was 0.4%, crude prevalence being 0% in children under five and 0.8% in those five and older. Geometric mean mf intensity among individuals with mf-positive night-bleed sample was 80 mf/ml (95% CI 18-343 mf/ml). There is strong evidence for a reduction in mf prevalence from 2007 to 2010, both among children under five years (p = 0.034) and in those aged five and above (p < 0.001).

Due to the small number of individuals with mf in 2010, risk factors were not evaluated for mf, but only for *W. bancrofti *antigen. Univariate risk factors for *W. bancrofti *antigen are detailed in Table 4.

**Table 4:** Univariate associations for *W. bancrofti *infection in 2010, with 95% confidence intervals (CI).

	N individuals	n (%) infected	OR	95% CI	Wald p
Sex (female)	701	23 (3.3)	0.78	0.44-1.39	0.403
Age category^1^:					
< 10	754	2 (0.3)	1.00	-	-
10-19	241	14 (5.8)	23.3	5.10-106.0	< 0.001
20-29	108	12 (11.1)	47.1	9.73-228.3	< 0.001
30-39	98	10 (10.2)	42.8	8.70-210.9	< 0.001
40-49	49	7 (14.3)	62.8	11.6-340.9	< 0.001
≥ 50	52	3 (5.8)	23.1	3.7-145.5	< 0.001
Socio-economic status^2^:					
Poorest	261	11 (4.2)	1.00	-	-
2^nd^	246	10 (4.1)	0.96	0.40-2.31	0.933
3^rd^	257	8 (3.1)	0.73	0.29-1.85	0.505
4^th^	271	5 (1.9)	0.43	0.15-1.25	0.110
Least poor	261	14 (5.4)	1.29	0.57-2.90	0.539
Village:					
Adyangoto B	174	12 (6.9)	1.46	0.64-3.34	0.364
Akabi	142	9 (6.3)	1.34	0.55-3.26	0.522
Okwor	150	4 (2.7)	0.54	0.17-1.71	0.289
Aridi	181	6 (3.3)	0.68	0.25-1.84	0.442
Alwar	226	1 (0.4)	0.09	0.01-0.69	0.004
Acapii	182	4 (2.2)	0.44	0.14-1.40	0.156
Adyangoto A^3^	249	12 (4.8)	1.00	-	-
Household size^4^:	1304	48 (3.7)	0.99	0.88-1.10	0.792
Main material of walls:					
Mud/dung	802	30 (3.7)	1.00	-	-
Brick	499	18 (3.6)	0.96	0.53-1.75	0.901
Main material of roof:					
Grass thatch	1007	36 (3.6)	1.00	-	-
Iron sheet	297	12 (4.0)	1.14	0.58-2.21	0.708
Toilet facilities:					
None	200	11 (5.5)	1.00	-	-
Pit latrine	1090	36 (3.3)	0.59	0.29-1.17	0.128
Education of head of household:					
None	131	5 (3.8)	1.00	-	-
Primary	982	40 (4.1)	1.07	0.41-2.76	0.889
Secondary or higher	190	2 (1.1)	0.27	0.05-1.42	0.096
Number nets in household (linear)	1304	48 (3.7)	1.05	0.79-1.40	0.748
Sleeping under net on previous night:					
Not sleeping under net	390	15 (3.9)	1.00	-	-
Sleeping under non-LLIN	204	12 (5.9)	1.56	0.72-3.41	0.258
Sleeping under LLIN	710	21 (3.0)	0.76	0.39-1.50	0.428
Universal net coverage in household^5^	350	8 (2.3)	0.53	0.25-1.15	0.110
Proportion of nets in household being LLINs	1304	48 (3.7)	0.99	0.98-1.00	0.008
Proportion of nets in household found hanging/tied over bed/mat	1304	48 (3.7)	0.99	0.98-1.00	0.105

^1 ^Initial categories of < 5 and 5-9 were found to be statistically similar, therefore combined into a single reference category;

^2 ^Socio-economic status estimated using principal component analysis based on ownership of key household assets [34];

^3 ^Adyangoto A village had largest denominator, therefore selected as reference category;

^4 ^Linear data is better fit for associations with infection than household size divided into quartiles;

^5 ^Universal coverage defined as households with at least one net for every two people in the household.

Two multivariate models were created; the first included individual use and type of mosquito nets, the second explored impact of universal net coverage in a household. No associations were found between individual use of untreated nets or LLINs and *W. bancrofti *antigen (data not shown). Universal household net coverage was found to be protective against *W. bancrofti *infection in the final multivariate model (OR = 0.44, 95% CI, 0.22-0.89, Table 5).

**Table 5:** Final multivariate associations between universal net coverage and *W. bancrofti *antigen in 2010, including all covariates retained in final models with 95% confidence intervals (CI).

	*W. bancrofti *(antigen)		
	**OR**	**95% CI**	**Wald p**

Universal net coverage^1^	0.44	0.22-0.89	0.022
Sex (female)	0.49	0.21-1.12	0.092
Age 10-19 years	27.56	6.22-121.92	< 0.001
Age 20-29 years	71.54	12.33-415.13	< 0.001
Age 30-39 years	49.07	12.37-194.66	< 0.001
Age 40-49 years	70.69	23.03-216.97	< 0.001
Age ≥ 50 years	26.51	2.62-268.26	0.005
Baseline infection prevalence in ≥ 5 yrs, by village^2^	1.04	0.93-1.17	0.471
Roof material: Iron sheet	-	-	-
Proportion of nets being LLINs	0.99	0.98-1.00	0.004
Toilet facilities: Pit latrine	0.37	0.12-1.16	0.088

Robust standard errors were applied to the final models.

Reference sex is male; reference age < 10 years; reference roof material grass thatch; reference toilet facility none.

^1 ^Universal net coverage defined as households with one or more net for every two people;

^2 ^Baseline village prevalence of *P. falciparum *among ≥ 5 years is included in *P. falciparum *models, while baseline village mf prevalence among ≥ 5 years is included in *W. bancrofti *models.

### *W. bancrofti *infection in mosquitoes

In 2007, 1,168 female *Anopheles *were caught and dissected. A total of nine were found to be carrying *W. bancrofti *larvae (0.77%, 95% CI: 0.35-1.46), with only two being infective L3 stages (0.17%, 95% CI: 0.02-0.62). Data specifying number of *Anopheles *caught per trap per night were not recorded in 2007, consequently, it was not possible to calculate the monthly biting rate and monthly transmission potential for the study period (July).

A total of 1,278 female *Anopheles *were caught and dissected in 2008, 467 *An. gambiae *and 811 *An. funestus*, and the estimated biting rate for the month of July was 304 bites/person/month. Prevalence of *W. bancrofti *in these mosquitoes was 1.8% (95% CI: 1.1-2.7), with prevalence in *An. gambiae *2.4% and *An. funestus *1.5%. Only one *An. gambiae *mosquito was found to have L3 *W. bancrofti *stages, giving an overall prevalence of infectivity in the *Anopheles *population of 0.08% (95% CI: 0.002-0.4), the monthly transmission potential for July was calculated to be 0.24 infectious bites/person/month.

In 2010, 2,959 female *Anopheles *were dissected, 828 *An. gambiae *and 2,131 *An. funestus*, which translated into a monthly biting rate of 540 bites/person/month for July. Three mosquitoes (0.1%, 95% CI: 0.02-0.3) were found to be infected with *W. bancrofti *larvae, all of which were L1 stage. Since no mosquito was found to have L3 *W. bancrofti*, the monthly transmission potential was zero. Mean larval count among infected mosquitoes was 2.0.

The proportion of mosquitoes infected with any stage of *W. bancrofti *larvae declined between 2007 and 2010 (Fisher's exact p = 0.001). Although there appeared to be a decrease in the proportion of mosquitoes with the infective L3 stage, this was not statistically significant (Fisher's exact p = 0.080). The biting rate increased from 2008 to 2010, while the transmission potential declined.

## Discussion

The aim of the current study was to investigate the impact of MDA on LF transmission where large-scale distribution of LLINs had been implemented. Over the three year follow-up period, it was found that coverage and use of LLINs increased immediately after a mosquito net distribution campaign, but declined over the next two years in the absence of access to replacement nets by the population. *W. bancrofti *mf prevalence declined sharply over the course of the study, to below 1%. Universal coverage of mosquito nets in a household was found to be protective against human *W. bancrofti *infection (based on antigen detection) in multivariate modelling. However, although there was a reduction in *W. bancrofti *infection prevalence in mosquitoes, there was an apparent increase in vector densities and monthly biting rates, suggesting there was limited protection from bites by *Anopheles *mosquitoes. This could suggest that the reduction in human *W. bancrofti *infection was primarily due to the MDA. Evidence from Papua New Guinea showed that one round of treatment with DEC alone can reduce mosquito infection levels [16]. Precisely determining the individual contributions of MDA and regular LLIN use to the observed reduction in *W. bancrofti *prevalence and transmission is, however, difficult due to inability to include a control group in the study design or adopt a factorial study design as withholding known efficacious interventions would have been unethical.

The impact of MDA in reducing population levels of *W. bancrofti *has been well documented [35-37], with a combination of ivermectin and albendazole appearing to give a faster but shorter-lived reduction in microfilaria rates than DEC and albendazole [38]. While a study in Kenya has demonstrated the reductions in infection levels can be maintained even when some rounds of MDA are missed [39], there is evidence for a waning effect of MDA after multiple rounds of treatment, resulting in residual low levels of infection in the population [37]. This decrease in effect of MDA after numerous rounds may be a result of incomplete coverage of the population, or the inadequate macrofilaricidal effect of drug combinations used. Other studies have shown that mosquito nets (both insecticide-treated and untreated) can reduce LF vector density and infectivity, hence making a significant contribution towards preventing LF transmission [20-24]. The most salient example of LF vector control comes from indoor residual spraying with DDT conducted at large-scale as part of malaria elimination efforts during the 1960s and 1970s. Vector control by means of this tool is thought to have brought about elimination of *W. bancrofti *from the Solomon Islands, where *An. punctualis *was the main vector [40]. In that setting, *W. bancrofti *was eliminated in the absence of MDA, purely as a collateral benefit of the ultimately unsuccessful malaria elimination activities. This has parallels with the current scale-up of ownership and use of LLINs in malaria endemic countries, which will likely impact on other mosquito-borne infections including LF. There remains a need for further evidence to establish the effectiveness of LLINs alone against LF, with promising results emerging from a study in a *L. loa*-endemic area of Nigeria [24], where MDA cannot be implemented and LLINs are therefore the primary control strategy.

Universal coverage with mosquito nets in a household was found to be the most important indicator of net ownership and use in this study, with a protective effect against *W. bancrofti *infection remaining in multivariate models. This indicator can be considered as an approximation to a household having a mosquito net for every sleeping space, although this was not measured directly in the current study. In addition to a household owning sufficient nets to cover all individuals, these nets must of course be in use. Throughout the study, a high proportion of all nets were found hanging or tied up in households, rather than being stored. In the current data, therefore, universal coverage can be assumed to mean that not only does a household own sufficient nets for all, but that all these nets are in regular use. The finding that mosquito net use on the night before the survey had no association with *W. bancrofti *infection, but universal coverage in a household was protective, perhaps represents the importance of consistent and long-term use of mosquito nets. This may also be related to the known inefficient transmission of *W. bancrofti*, with humans requiring multiple exposures before a productive infection develops.

The increase in mosquito biting rate between 2008 and 2010 indicates an increased vector population at the study site at the time of the survey, suggesting that LLINs afforded little protection. However, it should be noted that biting rate was not measured directly through human landing catches in this instance, but approximated during each survey using catches from CDC light traps. A variation in the timing of seasonal rains each year, and resultant changes in the peak of the mosquito population, is likely to have been a major contributor to the variation in mosquito population and biting rates observed between years. In the absence of monthly mosquito collection data this interpretation could, however, not be confirmed as we were unable to tell whether mosquito collections were always made at the same point in the annual population fluctuation. An alternative interpretation may be that a reduction in *W. bancrofti *transmission may have led to increased mosquito survival, as a result of facilitation [18], in turn increasing the overall mosquito population. However, a recent meta analysis of data documenting the effects of *W. bancrofti *infection on various mosquito species found limited evidence for any association [41], and it is therefore considered unlikely that the reduction in *W. bancrofti *transmission at the study site directly related to the increased mosquito population and biting rate. While the major indicator of effectiveness of LLINs in LF prevention is a reduction in filarial vector density [20], scale up in LLIN use has also been shown to reduce vector lifespan [42], which would consequently reduce the number of mosquitoes which live long enough for ingested mf to develop into infectious L3 stages, and would therefore reduce transmission. However, in the current study mosquito age was not evaluated, and should the increase in vector population indicated by these data hold true throughout the year, rather than for a single time point, then it would substantiate the suggestion that LLINs have contributed little to the reduction in *W. bancrofti *transmission.

A key strategy for defining the extent of current transmission is through entomological indicators. While dissection and examination of mosquitoes for presence of *W. bancrofti *larvae has long been considered the gold standard technique, molecular xenomonitoring has a number of advantages relevant to the LF elimination programme. We found that once *W. bancrofti *transmission had declined to very low levels, dissection of the large number of mosquitoes required to accurately determine transmission is laborious and time consuming. Resources permitting, investigators planning to conduct similar studies may choose to employ polymerase chain reaction (PCR) methods, which can reliably detect *W. bancrofti *from pooled samples of mosquitoes [43]. A modification of this PCR technique has been described that is able to detect the human-infective L3 larval stages in mosquitoes [44,45]. However, PCR does not allow for quantification of the intensity of infection and requires specialised equipment and highly trained staff. Nevertheless, PCR appears to be a useful method for validation purposes in the elimination programme.

The current study had a number of limitations. Most importantly it was not designed to determine the individual contributions of MDA and LLINs to the reduction in LF transmission. The baseline survey in July 2007 was implemented after MDA had already been conducted three times in the study area, in 2002, 2004 and 2005. Experience from other settings has shown that a single MDA round can reduce mf prevalence in the population by anything from 21% to 76%, depending on the transmission setting, while in years when no MDA is conducted a resurgence in mf prevalence may be observed [35,37,46]. It is therefore difficult to determine if the mf rates in 2007 are the true baseline for this setting, and to what extent the previous rounds of MDA and the two-year gap during which no MDA took place had reduced *W. bancrofti *infection in the human population. Furthermore, as experienced by other investigators, difficulties were encountered in determining the number MDA rounds that study participants had received [47]. Multivariate modelling was therefore not able to control for the number of times individuals had received albendazole plus ivermectin. It is possible that the observed protective effect of universal net coverage against *W. bancrofti *may have been confounded by those households and individuals being more health conscious and therefore more likely to have participated in multiple rounds of MDA.

The change in sampling strategy between baseline and final follow-up surveys presents a further limitation of this study. Baseline results found a lower than expected mf prevalence in the population, particularly in children under five years. In order to maximise the statistical power of the comparison in mf rates between baseline and follow-up and identify any recent change in *W. bancrofti *transmission, it was decided to increase the number of children under five tested in the 2010 survey. While this change in sampling means that crude population prevalence is not comparable between 2007 and 2010 data, age stratification to < 5 and ≥ 5, as well as calculation of age-adjusted prevalence has overcome this. Furthermore, use of ICTs as a primary diagnostic tool in 2010 allowed evaluation of *W. bancrofti *antigen prevalence, which is less affected by MDA than mf prevalence since ivermectin and albendazole have limited macrofilaricidal effect [48,49]. As transmission reduces it is antigen prevalence detected by ICTs, not mf detected by microscopy, that will indicate the continued presence of adult worms in the human population, which could lead to resurgence in transmission after MDA has ceased.

## Conclusions

This study has documented a sharp decline in *W. bancrofti *infection in humans, as well as reduction in transmission potential by the *Anopheles *vector, in a previously highly LF endemic area of Uganda.

The study area had received multiple rounds of MDA with albendazole and ivermectin, and seen a large increase in LLIN ownership and use. While statistical modelling did indicate some protective effect of universal coverage of LLINs against *W. bancrofti*, in this setting it was not possible to determine the effect of MDA and LLIN use against transmission individually. Furthermore, the impact of LLINs on transmission in mosquitoes is equivocal due to the observed increase in vector densities and monthly biting rates, despite a reduction a *W. bancrofti *prevalence in mosquitoes. The potential additional benefit of mosquito nets against LF should be taken into account in the current scale up of LLIN coverage for malaria prevention, as high population coverage of nets in areas where both malaria and LF are transmitted by anopheline mosquitoes could yield enhanced cost-effectiveness.

## Competing interests

The authors declare that they have no competing interests.

## Authors' contributions

RA contributed to study design, was responsible for project management in 2010, data analysis and interpretation, and developed the draft manuscript. DK was responsible for project management in 2007 and 2008. TO facilitated field activities and entomology work. AA led entomology and laboratory work. TE contributed to data analysis, interpretation, and revised the manuscript. SB and JK were responsible for study design, data interpretation and scientific guidance. All authors read and approved the final manuscript.
